# Use of intravascular ultrasound and long-term cardiac death or myocardial infarction in patients receiving current generation drug-eluting stents

**DOI:** 10.1038/s41598-022-12339-6

**Published:** 2022-05-17

**Authors:** Sang Yoon Lee, Ki Hong Choi, Young Bin Song, Taek Kyu Park, Joo Myung Lee, Jeong Hoon Yang, Jin-Ho Choi, Seung-Hyuk Choi, Hyeon-Cheol Gwon, Joo-Yong Hahn

**Affiliations:** 1 Division of Cardiology, Department of Internal Medicine, Heart Vascular Stroke Institute, Samsung Medical Center, Sungkyunkwan University School of Medicine, 81 Irwon-ro, Gangnam-gu, Seoul, 135-710 Republic of Korea; 2 Department of Critical Care Medicine, Samsung Medical Center, Sungkyunkwan University School of Medicine, Seoul, Republic of Korea; 3 Department of Emergency Medicine, Samsung Medical Center, Sungkyunkwan University School of Medicine, Seoul, Republic of Korea

**Keywords:** Cardiovascular biology, Interventional cardiology

## Abstract

Long-term follow-up data on differential effects of intravascular ultrasound (IVUS) according to lesion complexity are limited in patients undergoing percutaneous coronary intervention (PCI). The current study compared long-term clinical outcomes between IVUS-guided and angiography-guided PCI in patients with second-generation drug-eluting stents (DES). Between February 2008 and December 2015, 5488 patients undergoing PCI with second-generation DES were recruited from an institutional registry of Samsung Medical Center. The primary outcome was a composite of cardiac death or myocardial infarction (MI) during 46 months of median follow-up (interquartile range: 32–102 months). IVUS-guided PCI was performed in 979 patients (17.8%). IVUS-guided PCI was associated with a significantly lower risk of cardiac death or MI compared with angiography-guided PCI (5.7% vs. 12.9%, hazard ratio 0.408, 95% confidence interval 0.284–0.587, p < 0.001). Results were consistent after propensity score matching analysis with 801 matched pairs. In subgroup analysis, there was no significant interaction between lesion complexity (defined by complex procedures, P_interaction_ = 0.819, ACC/AHA lesion classification, P_interaction_ = 0.401 or SYNTAX score, P_interaction_ = 0.149) and use of IVUS for risk of cardiac death or MI. IVUS-guided second-generation DES implantation was associated with a significantly lower long-term risk of cardiac death or MI compared with angiography guidance, regardless of lesion complexity.

## Introduction

Intravascular ultrasound (IVUS) provides tomographic images of coronary artery structure and is a helpful tool for evaluating lesion geometry, reference vessel status, and post-intervention stent optimization during percutaneous coronary intervention (PCI)^1^. Several randomized trials conducted to identify the clinical benefits of IVUS-guided PCI compared with angiography-guided PCI have shown that IVUS guidance is associated with significantly lower risk of adverse events^2–5^. Despite clear evidence of the benefits of IVUS-guided PCI, it is not utilized frequently in real-world practice, especially for non-complex lesions^6–8^. This might be because previous studies have focused on the effects of IVUS use for complex lesion subsets, such as left main disease, chronic total occlusion, diffuse long lesion, or bifurcation lesion^3–5,9–11^.

Recently, the Intravascular Ultrasound Guided Drug Eluting Stents Implantation in All-Comers Coronary Lesion (ULTIMATE) randomized trial demonstrated that IVUS-guided drug-eluting stent (DES) implantation significantly reduced the risk of target vessel failure in all-comer patients with coronary artery disease (CAD)^2^. While a large prospective ADAPT-DES (The Assessment of Dual Antiplatelet Therapy With Drug-Eluting Stents) registry showed the benefits of IVUS were more prominent in patients with complex lesions^12^. Furthermore, some previous studies failed to show the significant clinical benefits of IVUS guidance in non-complex lesions^13–15^. However, there is a lack of data regarding the long-term follow-up outcomes of IVUS-guided PCI in patients with non-complex lesion.

Therefore, we sought to compare the long-term clinical outcomes between IVUS-guided versus angiography-guided PCI with second-generation DES in patients undergoing PCI and to identify whether the benefits of IVUS guidance differed according to lesion complexity.

## Methods

### Patient population and data collection

**Figure 1:**
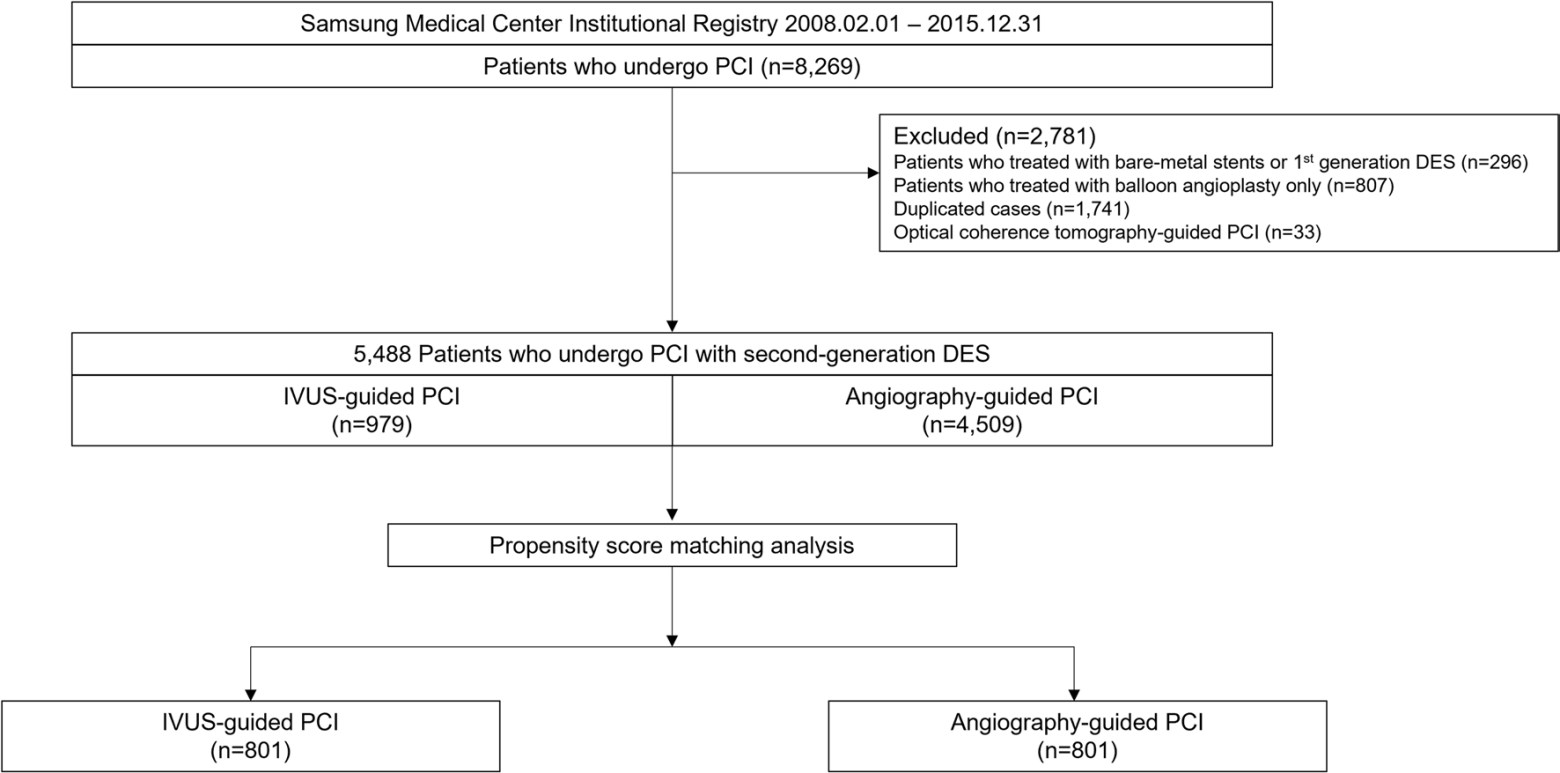
Study flow. A flow diagram is shown. DES, drug-eluting stent; IVUS, intravascular ultrasound; PCI, percutaneous coronary intervention.

This is a retrospective, single-center, observational study. From February 2008 to December 2015, a total of 5488 consecutive patients treated with PCI with second-generation DES was recruited from an institutional cardiovascular catheterization database of Samsung Medical Center (Clinicaltrials.gov, NCT03870815). Patients who treated with balloon angioplasty only, bare-metal stent (BMS), or first-generation DES were excluded. Patients who underwent PCI with optical coherence tomography guidance were also excluded from the current analysis (Fig. 1).

Baseline characteristics and clinical outcomes were collected prospectively from our PCI registry by research coordinators. Additional information was obtained through review of medical records or telephone contact, if necessary. The mortality data for patients who were lost to follow-up were confirmed by National Death Records. The study protocol was approved, and requirement for informed consent from individual patients was waived by the Institutional Review Board of Samsung Medical Center. This study was conducted according to the principles of the Declaration of Helsinki.

### Percutaneous coronary intervention and intravascular ultrasound

All interventions were performed according to current practice guidelines^16,17^. All study participants were prescribed the loading dose of aspirin (300 mg) and P2Y12 inhibitors (clopidogrel 300–600 mg, ticagrelor 180 mg, or prasugrel 60 mg) before PCI unless they had previously received these antiplatelet medications. Anticoagulation during PCI was performed using low-molecular weight heparin or unfractionated heparin to achieve an activated clotting time of 250 to 300 s. The revascularization treatment strategy, use of glycoprotein IIb/IIIa receptor inhibitors, choice of DES, duration of dual antiplatelet therapy, and any adjunctive pharmacologic treatment after PCI were at the discretion of the operators. IVUS imaging was performed using a commercially available system (Boston Scientific Corporation, San Jose, CA, USA or Volcano Corporation, Rancho Cordova, CA, USA) after administration of 0.2 mg intracoronary nitroglycerin. The use of IVUS during PCI was left to the operator’s preference. Timing of IVUS use was also based on operator discretion. The IVUS probe was pulled back automatically at a speed of 0.5 mm/s.

### Definitions and outcomes

The primary outcome of this study was a composite of cardiac death or myocardial infarction (MI) during follow-up. Secondary endpoints were cardiac death; all-cause death; MI; definite or probable stent thrombosis (ST); ischemia-driven target lesion revascularization (TLR); and major adverse cardiac events (MACE), a composite of cardiac death, MI, ST, and ischemia-driven TLR. Cardiac death was defined as death from any cardiac cause including sudden cardiac death, MI, congestive heart failure, and cardiac arrhythmias. MI was defined as an elevation of creatine kinase-myocardial band or troponin level greater than the upper limit of normal with concomitant ischemic symptoms or electrocardiography findings indicative of ischemia^18^. Peri-procedural MI was excluded as a clinical event in this study. Definite or probable ST was defined according to the definition of the Academic Research Consortium^19^. Ischemia-driven TLR was defined as a revascularization procedure at a previously treated segment from 5 mm proximal to the stent to 5 mm distal to the stent with ≥ 50% diameter stenosis and at least one of the following: (1) recurrence of angina, (2) positive non-invasive test, or (3) positive invasive physiologic test.

Anatomical lesion complexity was assessed using ACC/AHA lesion classification^20^, SYNTAX score (median value of 15)^21^, and previously used definition for complex PCI. (1) bifurcation lesion with side branch diameter ≥ 2.5 mm, (2) chronic total occlusion with duration ≥ 3 months, (3) unprotected left main CAD, (4) long lesion (implanted stent length ≥ 38 mm), (5) multi-vessel PCI (≥ 2 major epicardial coronary vessels treated at one PCI session), (6) multiple stent implantation (3 or more stents per patient), (7) in-stent restenosis lesion, or (8) severely calcified lesion (requiring a rotablator system)^9^.

### Statistical analysis

Differences between continuous variables were evaluated using Welch’s t-test, and the data are presented as mean ± standard deviation. Categorical data were analyzed between groups using the Chi-square test and are presented as number and relative frequency (%). The cumulative incidence of clinical events is presented as Kaplan–Meier estimates and compared using log-rank test. The Cox proportional hazards model was used to calculate hazard ratios (HR) with 95% confidence intervals (CI) to compare risk of clinical events between IVUS-guided versus angiography-guided PCI groups. In the multivariable model, we included covariates that were significant in univariate analysis or that were clinically relevant. The adjusted HRs and 95% CIs were evaluated by Cox regression to identify independent predictors of clinical events based on old age (> 65 years), male, hypertension, diabetes mellitus, hyperlipidemia, chronic kidney disease, current smoker, previous history of PCI, MI, or cerebrovascular accident, reduced left ventricular ejection fraction (< 40%), acute coronary syndrome, multivessel disease, left main disease, lesion locations (LAD, LCX, RCA), complex procedure, and medications (aspirin, P2Y12 inhibitor, statin, beta-blocker, and renin-angiotensin receptor blocker).

To adjust for concomitant baseline demographics, cardiovascular risk factors, clinical presentation, and lesion severity, we performed propensity score matching analysis. A total of 801 matched pairs was generated. A matched population was obtained by logistic regression and one-to-one nearest neighbor matching based on propensity scores. A full non-parsimonious model was developed with all variables listed in Tables 1 and 2, except procedural characteristics, which might be affected by IVUS use. After matching by propensity score, the covariate balance was evaluated using standardized mean differences. Standardized mean differences were limited to within ± 10% across all matched covariates for successful balance between the two groups. Continuous variables were compared using the paired t-test or Wilcoxon signed-ranked test, as appropriate. Categorical variables were compared with McNemar’s or Bowker’s test of symmetry, as appropriate. Cumulative incidence rates of adverse events were estimated by the Kaplan–Meier method and compared by the paired Prentice-Wilcoxon test. Cox proportional hazard models were used to compare outcomes in matched groups.

**Table 1:** Baseline characteristics according to use of IVUS in overall and propensity matched populations.

	Overall population (n = 5488)				Propensity-matched population (n = 1602)			
	IVUS-guided (n = 979)	Angiography-guided (n = 4,509)	p value	SMD, %	IVUS-guided (n = 801)	Angiography-guided (n = 801)	p value	SMD, %
**Demographics**								
Age, years	62.2 ± 10.5	64.1 ± 11.1	< 0.001	− 12.5	62.2 ± 10.5	62.8 ± 11.2	0.243	1.5
Male	772 (78.9)	3384 (75.1)	0.013	9.3	621 (77.5)	628 (78.4)	0.718	− 2.1
**Cardiovascular risk factors**								
Hypertension	570 (58.2)	2752 (61.0)	0.111	− 5.7	472 (58.9)	482 (60.2)	0.647	− 2.5
Diabetes mellitus	459 (46.9)	2450 (54.3)	< 0.001	− 14.9	381 (47.6)	388 (48.4)	0.764	− 1.8
Chronic kidney disease	55 (5.6)	396 (8.8)	0.001	− 13.7	51 (6.4)	56 (7.0)	0.689	− 2.8
Hyperlipidemia	325 (33.2)	1546 (34.3)	0.539	− 2.3	267 (33.3)	272 (34.0)	0.832	− 1.3
Current smoker	182 (18.6)	1038 (23.0)	0.003	− 11.4	160 (20.0)	150 (18.7)	0.569	3.2
Obesity (BMI > 25 kg/m^2^)	395 (40.4)	1934 (42.9)	0.154	− 5.2	324 (40.5)	349 (43.6)	0.224	− 6.4
Previous PCI	165 (16.9)	669 (14.8)	0.123	5.4	133 (16.6)	134 (16.7)	1.000	− 0.3
Previous MI	94 (9.6)	469 (10.4)	0.272	− 2.7	78 (9.7)	82 (10.2)	0.803	− 1.7
Previous CVA	51 (5.2)	288 (6.4)	0.189	− 5.3	43 (5.4)	39 (4.9)	0.734	2.2
Peripheral artery disease	28 (2.9)	111 (2.5)	0.544	2.4	25 (3.1)	26 (3.3)	1.000	− 0.7
**Initial presentation**								
LVEF, %	60.8 ± 9.2	59.5 ± 9.8	< 0.001	12.7	60.5 ± 9.4	60.4 ± 9.0	0.736	1.1
Acute coronary syndrome	337 (34.4)	2066 (45.8)	< 0.001	− 24.0	284 (35.5)	266 (33.2)	0.371	4.7
Cardiogenic shock	1 (0.1)	16 (0.4)	0.331	− 6.0	1 (0.1)	4 (0.5)	0.370	− 7.3
**Medications**								
Aspirin	907 (92.7)	4110 (91.2)	0.147	5.7	743 (92.8)	723 (90.3)	0.089	9.6
P2Y12 inhibitors^a^	920 (94.0)	4235 (93.9)	1.000	0.2	756 (94.4)	756 (94.4)	1.000	0.0
Statin	908 (92.8)	4099 (90.9)	0.074	7.1	738 (92.1)	37 (92.0)	1.000	0.5
Beta-blocker	430 (43.9)	2112 (46.8)	0.104	− 5.9	347 (43.0)	355 (44.3)	0.763	− 1.8
ACE inhibitor or ARB	463 (47.3)	2280 (50.6)	0.069	− 6.6	386 (48.2)	360 (44.9)	0.211	6.5

ACE, angiotensin converting enzyme; ARB, angiotensin receptor blocker; BMI, body mass index; CVA, cerebrovascular accident; IVUS, intravascular ultrasound; LVEF, left ventricular ejection fraction; MI, myocardial infarction; PCI, percutaneous coronary intervention; SMD, standardized mean difference.

^a^P2Y12 inhibitors were clopidogrel, ticagrelor, or prasugrel.

**Table 2:** Angiographic characteristics according to use of IVUS in overall and propensity-matched populations.

	Overall population (n = 5488)				Propensity-matched population^c^ (n = 1602)			
	IVUS-guided (n = 979)	Angiography-guided (n = 4509)	p value	SMD, %	IVUS-guided (n = 801)	Angiography-guided (n = 801)	p value	SMD, %
**Lesion characteristics**								
Multivessel disease	613 (62.6)	2599 (57.6)	0.005	10.3	464 (57.9)	468 (58.4)	.879	− 1.0
Lesion location (per vessel)								
Left main	260 (26.6)	123 (2.7)	< 0.001	53.9	118 (14.7)	105 (13.1)	0.386	3.7
LAD	825 (84.3)	3265 (72.4)	< 0.001	32.6	672 (83.9)	691 (86.3)	0.207	− 6.5
LCX	417 (42.6)	2327 (51.6)	< 0.001	− 18.2	336 (42.0)	352 (44.0)	0.449	− 4.0
RCA	366 (37.4)	2432 (53.9)	< 0.001	− 34.2	318 (39.7)	335 (41.8)	0.416	− 4.4
SYNTAX score	14.7 ± 6.4	13.0 ± 5.9	< 0.001	26.2	14.4 ± 6.2	14.5 ± 6.5	0.584	− 2.7
Type B2/C^a^	825 (84.3)	3153 (69.9)	< 0.001	39.4	659 (82.3)	668 (83.4)	0.596	− 3.1
Complex procedure^b^	839 (85.7)	2396 (53.1)	< 0.001	93.0	661 (82.5)	652 (81.4)	0.603	3.2
Bifurcation lesion	572 (58.4)	754 (16.7)	< 0.001	84.6	407 (50.8)	386 (48.2)	0.318	5.3
Chronic total occlusion	141 (14.4)	575 (12.8)	0.181	4.7	127 (15.9)	147 (18.4)	0.207	− 7.1
Long lesion (stent ≥ 38 mm)	443 (45.3)	1252 (27.8)	< 0.001	35.1	356 (44.4)	363 (45.3)	0.763	− 1.8
Multi-vessel PCI	373 (38.1)	1278 (28.3)	< 0.001	20.0	267 (33.3)	289 (36.1)	0.270	− 5.6
Multiple stent implantation (≥ 3)	156 (15.9)	405 (9.0)	< 0.001	19.0	121 (15.1)	141 (17.6)	0.199	− 6.8
In-stent restenosis	54 (5.5)	231 (5.1)	0.673	1.7	46 (5.7)	42 (5.2)	0.742	2.1
Heavy calcification	42 (4.3)	59 (1.3)	< 0.001	14.7	34 (4.2)	39 (4.9)	0.632	− 3.1
**Procedural characteristics**^**c**^								
Successful PCI	962 (98.3)	4350 (96.5)	0.005	13.7	784 (97.9)	782 (97.6)	0.866	1.9
Trans-radial approach	774 (79.1)	3624 (80.4)	0.374	− 3.2	634 (79.2)	652 (81.4)	0.286	− 5.5
Total lesion length	32.9 ± 26.4	27.9 ± 20.4	< 0.001	19.2	32.8 ± 26.7	33.9 ± 24.7	0.382	− 4.2
Number of treated lesions	2.4 ± 1.5	2.4 ± 1.5	0.528	2.3	2.4 ± 1.4	2.4 ± 1.5	0.365	− 4.5
Implanted stent number	1.6 ± 0.9	1.4 ± 0.8	< 0.001	23.5	1.6 ± 0.9	1.6 ± 1.0	0.697	0.0
Mean stent diameter, mm	3.2 ± 0.5	3.0 ± 0.6	< 0.001	36.2	3.2 ± 0.5	3.0 ± 0.5	< 0.001	
Total stent length, mm	41.4 ± 24.7	35.8 ± 21.5	< 0.001	24.2	41.2 ± 24.8	42.9 ± 26.2	0.190	
Maximum balloon pressure, mmHg	15.6 ± 3.2	14.3 ± 3.8	< 0.001	37.0	15.5 ± 3.4	14.6 ± 3.9	< 0.001	
Adjunctive balloon dilatation	425 (43.4)	711 (15.8)	< 0.001	63.4	314 (39.2)	182 (22.7)	< 0.001	
Timing of IVUS use	979 (100)	0 (0)	< 0.001		801 (100)	0 (0)	< 0.001	
Pre- and post-stent	701 (71.6)				555 (69.3)			
Pre-PCI only	105 (10.7)				99 (12.3)			
Post-stent only	173 (17.7)				147 (18.4)			

ACC, American College of Cardiology; AHA, American Heart Association; IVUS, intravascular ultrasound; LAD, left anterior descending artery; LCX, left circumflex artery; RCA, right coronary artery; SMD, standardized mean difference; SYNTAX, Synergy between PCI with Taxus and Cardiac Surgery.

^a^AHA/ACC lesion classification.

^b^Complex procedure was defined as bifurcation, chronic total occlusion, left main disease, long lesion, multivessel PCI, multiple stent implantation, in-stent restenosis, or heavily calcified lesion.

^c^Procedural characteristics that might be affected by IVUS use were excluded from the propensity score matched model.

All probability values were two-sided, and p-values < 0.05 were considered statistically significant. Statistical analyses were performed using R Statistical Software (version 3.6.0; R Foundation for Statistical Computing, Vienna, Austria)^22^.

## Results

### Baseline clinical, angiographic, and procedural characteristics

Among the total 5488 patients who underwent PCI with current generation DES, 979 (17.8%) patients received PCI with IVUS guidance. Patients undergoing PCI with angiography guidance were older and more likely to have general cardiovascular risk factors including hypertension, diabetes mellitus, hyperlipidemia, chronic kidney disease, and current smoker compared to those with IVUS guidance (Table 1). The proportion of patients presenting acute coronary syndrome at the index procedure was significantly higher in the angiography-guided PCI group than in the IVUS-guided PCI group. However, there was no significant difference in the proportion of cardiogenic shock at presentation. Left ventricular ejection fraction was significantly lower in the angiography-guided PCI group. Table 2 shows lesion and procedural characteristics according to use of IVUS. Compared to patients who underwent PCI with angiography guidance, those with IVUS guidance were more likely to have multivessel disease and left main disease. The IVUS-guided PCI group showed a higher mean implanted number of stents, larger implanted stent diameter, longer total stent length, and higher maximum balloon pressure compared with the angiography-guided PCI group. In the IVUS-guided PCI group, IVUS was used only before PCI in 105 patients (10.7%), only post-stent in 173 patients (17.7%), and both pre- and post-stent in 701 patients (71.6%) (Table 2). After propensity score matching, baseline clinical and angiographic differences according to IVUS usage were well balanced (Tables 1 and 2).

### Proportion of IVUS-guided PCI in real-world practice

**Figure 2:**
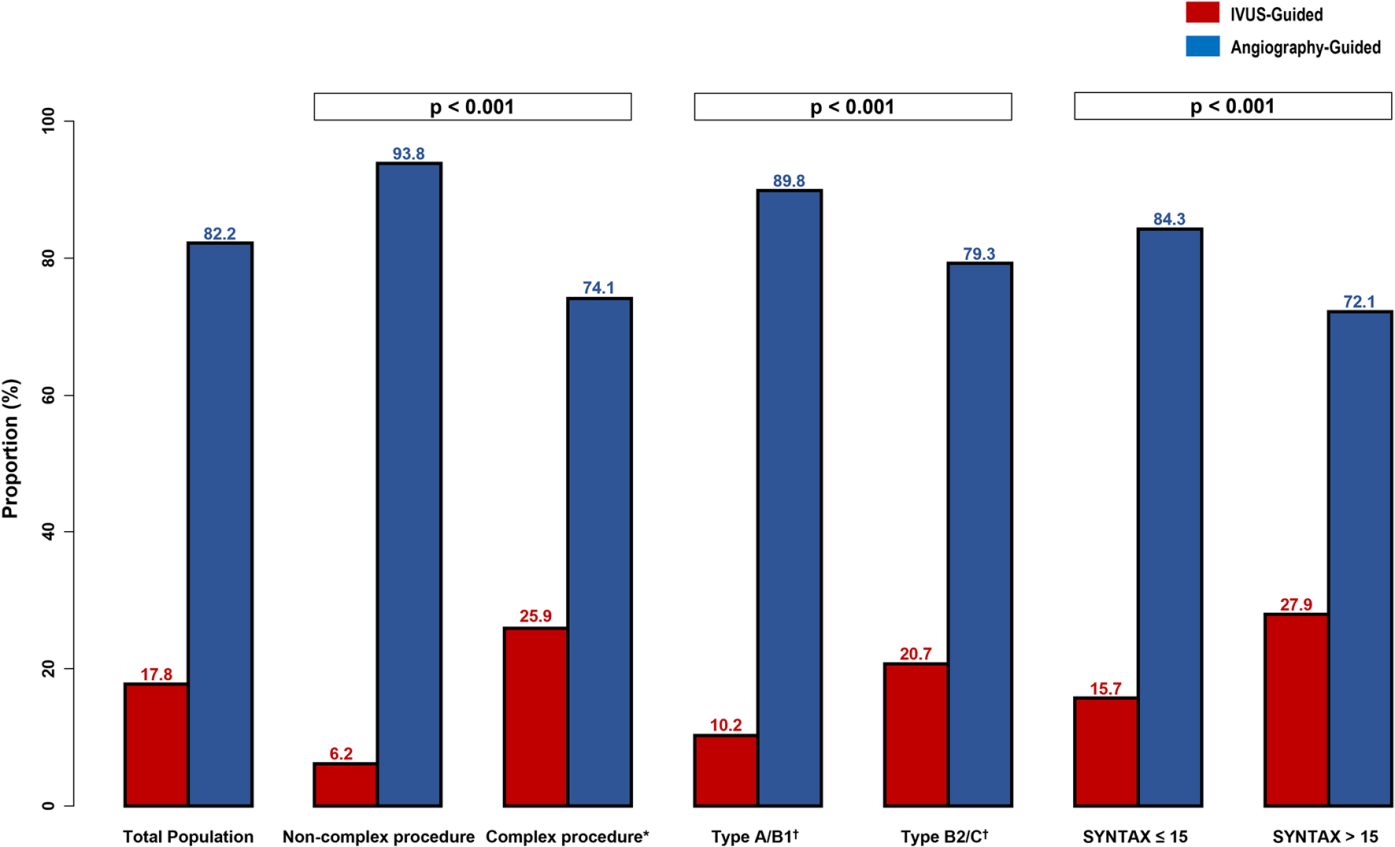
Proportion of IVUS-guided PCI according to lesion complexity. The bar chart shows proportion of IVUS-guided PCI according to lesion complexity. The proportion of IVUS-guided PCI (red bar) versus angiography-guided PCI (blue bar) are shown. *Complex procedure is defined as bifurcation, chronic total occlusion, left main disease, long lesion, multivessel PCI, multiple stent implantation, in-stent restenosis, or heavily calcified lesion. ^†^AHA/ACC lesion classification. IVUS, intravascular ultrasound; PCI, percutaneous coronary intervention; SYNTAX, Synergy between PCI with Taxus and Cardiac Surgery.

Patients who required complex procedures tended to comprise a higher proportion of IVUS-guided PCI than those who required non-complex procedures (25.9% vs. 6.2%, p < 0.001) (Fig. 2). Similarly, the proportion of IVUS-guided PCI was significantly higher in patients with type B2/C lesion (type B2/C vs. type A/B1, 20.7% vs. 10.2%, p < 0.001) or high SYNTAX score (SYNTAX score > 15 vs. SYNTAX score $\le$ 15, 27.9% vs. 15.7%, p < 0.001) than those with type A/B1 lesion or low SYNTAX score.

### Clinical outcomes

**Figure 3:**
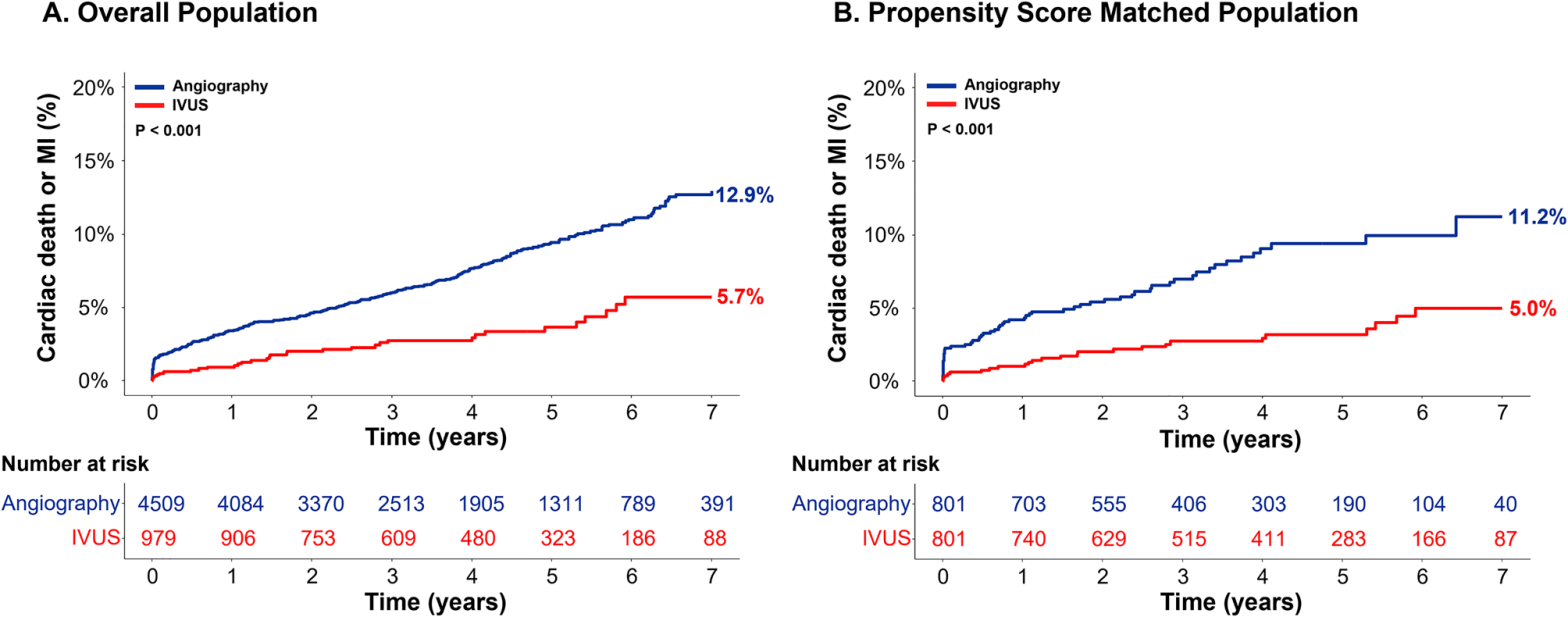
Comparison of primary composite endpoint between IVUS-guided PCI versus angiography-guided PCI in overall and propensity score matched population. The Kaplan–Meier survival curve is shown for the comparison of primary composite endpoint (cardiac death or MI) between IVUS-guided PCI (red line) and angiography-guided PCI (blue line) in overall (**A**) and propensity-matched population (**B**). IVUS, intravascular ultrasound; MI, myocardial infarction; PCI, percutaneous coronary intervention.

The median follow-up duration was 46 months (interquartile range from 32 to 102). A total of 373 cardiac deaths or MIs occurred during the study period, and the cumulative incidence of the primary composite endpoint was significantly lower in the IVUS-guided PCI group than in the angiography-guided PCI group (5.7% vs 12.9%, HR 0.408, 95% CI 0.284–0.587, p < 0.001) (Fig. 3 and Table 3). The risks of cardiac death (4.9% vs. 11.7%, HR 0.388, 95% CI 0.260–0.580, p < 0.001), all-cause death (8.3% vs. 16.3%, HR 0.480, 95% CI 0.357–0.645, p < 0.001), MI (2.1% vs. 3.4%, HR 0.439, 95% CI 0.236–0.816, p = 0.009) and MACE (9.0% vs. 15.9%, HR 0.577, 95% CI 0.436–0.762, p < 0.001) were significantly lower in the IVUS-guided PCI group (Table 3 and Supplementary Fig. 1). A difference in MI between the two groups is mainly driven by the difference in target vessel MI rather than in non-target vessel MI (Table 3). After 1:1 matching by propensity score, the risk of cardiac death or MI remained significantly lower in patients treated with IVUS-guided PCI than in those with angiography-guided PCI (5.0% vs. 11.2%, HR_matched_ 0.368, 95% CI 0.230–0.587, p < 0.001) among 801 matched pairs (Fig. 3 and Table 3). In multivariable analysis, IVUS-guided PCI showed an independent protective effect against cardiac death or MI. Other independent predictors were type B2/C of AHA/ACC lesion classification, old age (> 65 years), diabetes mellitus, chronic kidney disease, previous history of MI, reduced left ventricular ejection fraction (< 40%), acute coronary syndrome, and left main disease (Table 4).

**Table 3:** Long-term clinical outcomes of IVUS-guided PCI versus angiography-guided PCI.

	IVUS-guided (N = 979)	Angiography-guided (N = 4,509)	Univariate analysis		Multivariate analysi^a^		Propensity matching analysis	
			HR (95% CI)	p value	HR (95% CI)	p value	HR (95%CI)	p value
Cardiac death or MI	32 (5.7%)	341 (12.9%)	0.408 (0.284–0.587)	< 0.001	0.385 (0.261–0.568)	< 0.001	0.368 (0.230–0.587)	< 0.001
Cardiac death	26 (4.9%)	292 (11.7%)	0.388 (0.260–0.580)	< 0.001	0.389 (0.253–0.598)	< 0.001	0.387 (0.234–0.639)	< 0.001
**MI**	11 (2.1%)	111 (3.4%)	0.439 (0.236–0.816)	0.009	0.306 (0.157–0.597)	< 0.001	0.289 (0.130–0.642)	0.002
Target vessel MI	6 (1.1%)	63 (1.9%)	0.423 (0.183–0.976)	0.044	0.219 (0.090–0.535)	< 0.001	0.189 (0.072–0.497)	< 0.001
Non-target vessel MI	5 (1.0%)	48 (1.4%)	0.461 (0.184–1.158)	0.099	0.512 (0.185–1.422)	0.199	2.591 (0.269–24.99)	0.410
All-cause death	49 (8.3%)	446 (16.3%)	0.480 (0.357–0.645)	< 0.001	0.492 (0.358–0.676)	< 0.001	0.465 (0.319–0.677)	< 0.001
Stent thrombosis	18 (3.0%)	74 (3.0%)	1.065 (0.636–1.782)	0.812	0.253 (0.110–0.583)	0.001	0.309 (0.132–0.723)	0.007
Ischemia-driven TLR	7 (0.7%)	68 (1.7%)	0.466 (0.214–1.015)	0.054	0.733 (0.418–1.287)	0.279	0.875 (0.427–1.792)	0.714
MACE^b^	56 (9.0%)	423 (15.9%)	0.577 (0.436–0.762)	< 0.001	0.545 (0.404–0.736)	< 0.001	0.583 (0.405–0.840)	0.004

CI, confidence interval; HR, hazard ratio; IVUS, intravascular ultrasound; MACE, major adverse cardiac event; MI, myocardial infarction; PCI, percutaneous coronary intervention; TLR, target-lesion revascularization.

^a^Adjusted variables were age; sex; hypertension; diabetes mellitus; hyperlipidemia; chronic kidney disease; current smoker; previous history of PCI, MI, or cerebrovascular accident; left ventricular ejection fraction; acute coronary syndrome; multivessel disease; left main disease; lesion locations; complex procedure; and medications (aspirin, P2Y12 inhibitor, statin, beta-blocker, and renin-angiotensin receptor blocker).

^b^MACE was defined as: a composite of cardiac death, MI, stent thrombosis, and ischemia driven TLR.

**Table 4:** Independent predictors of cardiac death or myocardial infarction after PCI.

	Adjusted HR^a^	95% CI	p value
IVUS-guided PCI	0.385	0.261–0.568	< 0.001
AHA/ACC lesion classification B2/C	1.518	1.156–1.994	0.003
Age > 65 years	2.209	1.709–2.856	< 0.001
Diabetes mellitus	1.637	1.299–2.063	< 0.001
Chronic kidney disease	2.717	2.122–3.478	< 0.001
History of MI	1.535	1.156–2.040	0.003
Left ventricular ejection fraction < 40%	2.898	2.218–3.788	< 0.001
Acute coronary syndrome	1.842	1.477–2.296	< 0.001
Left main disease	2.654	1.855–3.797	< 0.001

ACC, American College of Cardiology; AHA, American Heart Association; CI, confidence interval; HR, hazard ratio; IVUS, intravascular ultrasound; MACE, major adverse cardiac event; MI, myocardial infarction; PCI, percutaneous coronary intervention; TLR, target-lesion revascularization.

^a^Adjusted variables were use of IVUS; age; sex; hypertension; diabetes mellitus; hyperlipidemia; chronic kidney disease; current smoker; previous history of PCI, MI, or cerebrovascular accident; left ventricular ejection fraction; acute coronary syndrome; multivessel disease; left main disease; lesion location; complex procedure; and medications (aspirin, P2Y12 inhibitor, statin, beta-blocker, and renin-angiotensin receptor blocker).

To explore the beneficial effects of IVUS guidance on both short-term and long-term follow-up outcomes, a landmark analysis was performed at the time point of 1-year. Differences in the rates of cardiac death or MI were prominent within 1 year after the index PCI, with continued divergence of the curves throughout the study period (Supplementary Fig. 2).

### Clinical outcomes according to lesion or clinical complexity

**Figure 4:**
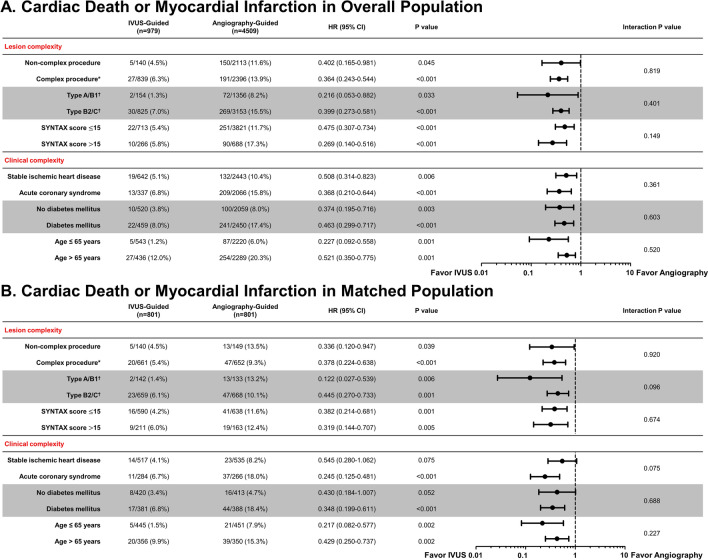
Subgroup analysis according to lesion complexity and clinical complexity. A forest plot shows the subgroup analysis with comparative hazard ratio and 95% confidence interval of cardiac death or MI in overall population (**A**) and matched population (**B**) between IVUS- versus angiography-guided PCI. *Complex procedure is defined as bifurcation, chronic total occlusion, left main disease, long lesion, multivessel PCI, multiple stent implantation, in-stent restenosis, or heavily calcified lesion. ^†^AHA/ACC lesion classification. CI, confidence interval; HR, hazard ratio; IVUS, intravascular ultrasound; PCI, percutaneous coronary intervention.

To explore the differential effects of IVUS-guided PCI compared with angiography-guided PCI according to lesion or clinical complexity, a subgroup analysis was performed in both overall and matched population. There was no significant difference in the relationship between use of IVUS and cardiac death or MI according to complex procedures during PCI (interaction p = 0.819), ACC/AHA classification (B2/C vs. A/B1, interaction p = 0.401), or SYNTAX score (≤ 15 vs. > 15, interaction p = 0.149), which was also consistent in matched population (Fig. 4). In addition, the beneficial effects of IVUS-guided PCI on clinical outcomes were consistent according to clinical complexity (acute coronary syndrome, diabetes mellitus, or old age) (Fig. 4).

## Discussion

The current study compared long-term clinical outcomes between IVUS guidance versus angiography guidance in patients with CAD who underwent PCI with second-generation DES, and the principal findings are as follows. (1) In real-world practice, use of IVUS tended to be more frequent when performing PCI for complex lesions. (2) Compared with angiography-guided PCI, IVUS-guided PCI with second-generation DES was strongly associated with lower risk of cardiac death or MI in patients with CAD. (3) The effects of IVUS guidance during PCI were identified regardless of lesion complexity or clinical complexity.

Although IVUS is well-known as a useful tool for evaluation of lesion characteristics and optimization of PCI procedures, especially in complex lesion subsets, the beneficial effects of IVUS guidance for long-term outcomes in routine clinical practice are controversial. Most previous studies comparing outcomes between IVUS- and angiography-guided PCI have been focused on complex lesion subsets, such as left main disease, bifurcations, chronic total occlusions, or diffuse long lesions^3–5,9,23,24^. In addition, previous randomized trials showed lower adverse cardiac events with IVUS-guided PCI, but did not show the improvement in hard endpoints, such as cardiac death or MI^2–5^. In this regard, the current guidelines recommend that IVUS could be considered in selected patients, including those with left main disease or in-stent restenosis^16,17^. In line with such guidelines, the current study showed that use of IVUS was more frequent during complex PCI in real-world practice.

After introduction of DES, which is associated with dramatic reductions of stent-related adverse events, two large non-randomized prospective registries and one randomized controlled trial were conducted for evaluating clinical benefits of routine use of IVUS guidance and consistently showed that IVUS-guided PCI was associated with significantly lower risk of 1- or 2-year adverse events compared with angiography-guided PCI in patients^2,12,25^. Recently, Mentias et al. reported the long-term beneficial effect of IVUS-guided PCI in patients by using the Medicare system with extremely large study population^26^. However, in that study, more than 20 percent of the study population have received PCI with BMS, which is not currently used, and the details of procedural or lesion characteristics are lacking due to the limitation of the study design. Therefore, we sought to compare the long-term clinical outcomes between IVUS- and angiography-guided PCI in patients treated with the second-generation DES by using the large institutional registry, which can contain detailed information about the procedural or lesion characteristics. In agreement with previous results, we found that routine IVUS-guided PCI with second-generation DES was associated with significantly reduced risks of cardiac death or MI during a median 46 months of follow-up compared with angiography-guided PCI in patients, even after strict adjustments for baseline differences using propensity score matching and multivariable Cox regression analysis. In addition, the IVUS-guided PCI group had a larger stent size, longer stent length, higher proportion of post-dilatation, and higher inflation pressures, which are surrogate markers of stent optimization^27^. These results support the hypothesis that routine use of IVUS is helpful to optimize stent deployment and to reduce long-term stent-related adverse events in patients with CAD who underwent PCI with second-generation DES.

Coronary artery lesion complexity is a powerful indicator of poor clinical outcomes in patients undergoing PCI^28^. In this regard, use of IVUS during PCI, which requires extra cost and time, remains debatable when treating non-complex coronary lesion subsets, where minimal clinical benefits are expected from a procedure. A previous study demonstrated that complex IVUS-guided PCI was associated with significantly reduced risk of adverse events up to 10 years (defined as bifurcation lesion, chronic total occlusion, unprotected left main disease, long lesion, multi-vessel PCI, multiple stent implantation, in-stent restenosis lesion, or severely calcified lesion)^9^. However, there are limited data regarding the efficacy and safety of IVUS guidance in patients with non-complex lesions who underwent PCI with DES. Although the ADAPT-DES registry and the ULTIMATE randomized trial consistently showed the beneficial effects of IVUS-guided PCI even in patients with non-complex lesions, there were discrepancies in the interaction between lesion complexity and use of IVUS for clinical outcomes among the two studies, and these studies reported only 1-year outcomes. Surprisingly, recent large Medicare based registry shows the benefits of IVUS guidance was significantly greater in the non-complex PCI than in the complex PCI with significant interaction (p < 0.001)^26^. On the other hand, there was no significant difference in 1-year MACE between IVUS- and angiography-guided PCI for short-length lesions in one observational study^14^. Because of these conflicting results, we additionally performed a subgroup analysis to determine whether the long-term benefits of IVUS differs according to type of lesion complexity. Interestingly, use of IVUS was associated with a significantly lower risk of cardiac death or MI in both complex and non-complex procedure groups. Similarly, when we classified lesion complexity using the AHA/ACC lesion classification (Type A/B1 vs. Type B2/C) or the SYNTAX score (≤ 15 vs. > 15), PCI with IVUS guidance showed favorable long-term outcomes regardless of lesion complexity. Taken together, our results suggest that routine use of IVUS might be helpful to reduce future adverse events even when performing PCI with non-complex lesions.

In contrast to a previous study^9^, routine IVUS-guided PCI did not show a significant difference in rates of ischemia-driven TLR compared with angiography-guided PCI. A possible explanation of this discrepancy is the inclusion of patients with non-complex lesions in the current study. In addition, unlike previous studies, the current study population received PCI with second-generation DES only. Therefore, long-term follow-up data for randomized controlled trials comparing outcomes between IVUS-guided and angiography-guided PCI for patients treated with current generation DES will be needed to confirm our findings.

The main limitation of this study is that it is a retrospective, single-center, and observational study. Although selection bias affecting the independent variables was balanced by propensity score matching, the use of a matched population has fundamental limitations compared with real randomized clinical trials. In this regard, the current study was a hypothesis-generating study, and the results should be interpreted with caution. Due to limited clinical evidence, the proportion of IVUS-guided PCI in patients with non-complex lesions was relatively small compared to that of IVUS guidance in patients with complex lesions. This low proportion of IVUS usage could have masked the real effects of IVUS guidance, especially in non-complex lesions. Among patients with IVUS guidance, no definitive criteria for IVUS optimization were suggested. Despite IVUS guidance, some patients might not have experienced optimal stent deployment or expansion and might have experienced poor clinical outcomes. Because of the definition and data collection process of endpoints, the significant difference of some endpoints between IVUS- and angiography-guided group could be underestimated. The incidence of ischemia-driven TLR, which could be identified only in few patients with evidence of myocardial ischemia, was relatively lower, compared to other endpoints. If patients were lost to follow-up, mortality data with cause were obtained through a National Death Records. On the other hand, other endpoints could not be investigated in loss of follow-up patients due to the limitations of registry data. These might affect the higher rates of mortality and relatively lower rates of other clinical endpoints.

In conclusion, IVUS guidance during PCI with second-generation DES showed significantly lower long-term risks of cardiac death and MI in patients with CAD compared with angiography guidance only. These results suggest that routine use of IVUS would actively be considered to reduce the long-term risk of adverse events after PCI, regardless of lesion complexity.

## Supplementary Information

Supplementary Figures.

## Abbreviations

CI: Confidence interval
DES: Drug-eluting stent
HR: Hazard ratio
IVUS: Intravascular ultrasound
MACE: Major adverse cardiac event
MI: Myocardial infarction
ST: Stent thrombosis
TLR: Target lesion revascularization
