# Supplementary material for: Use of intravascular ultrasound and long-term cardiac death or myocardial infarction in patients receiving current generation drug-eluting stents
Source: Sci Rep. 2022 May 17;12:8237. doi: 10.1038/s41598-022-12339-6 (PMC9114360; doi:10.1038/s41598-022-12339-6)
Supplement: Supplementary file 1 — Supplementary Figures. [file 41598_2022_12339_MOESM1_ESM.docx]

**Supplementary Appendix**

**Use of Intravascular Ultrasound and Long-term Cardiac Death or Myocardial Infarction in Patients receiving Current Generation Drug-eluting Stents**

Sang Yoon Lee, MD^✝^; Ki Hong Choi, MD, PhD^✝^; Young Bin Song, MD, PhD*; Taek Kyu Park, MD, PhD; Joo Myung Lee, MD, MPH, PhD; Jeong Hoon Yang, MD, PhD; Jin-Ho Choi, MD, PhD; Seung-Hyuk Choi, MD, PhD; Hyeon-Cheol Gwon, MD, PhD; Joo-Yong Hahn, MD, PhD*

**Table of Contents**

**(1) Supplementary Fig. 1**

**(2) Supplementary Fig. 2**

**Supplementary Fig. 1. Comparison of Clinical Outcomes between IVUS-guided PCI and Angiography-guided PCI with Second-generation DES**


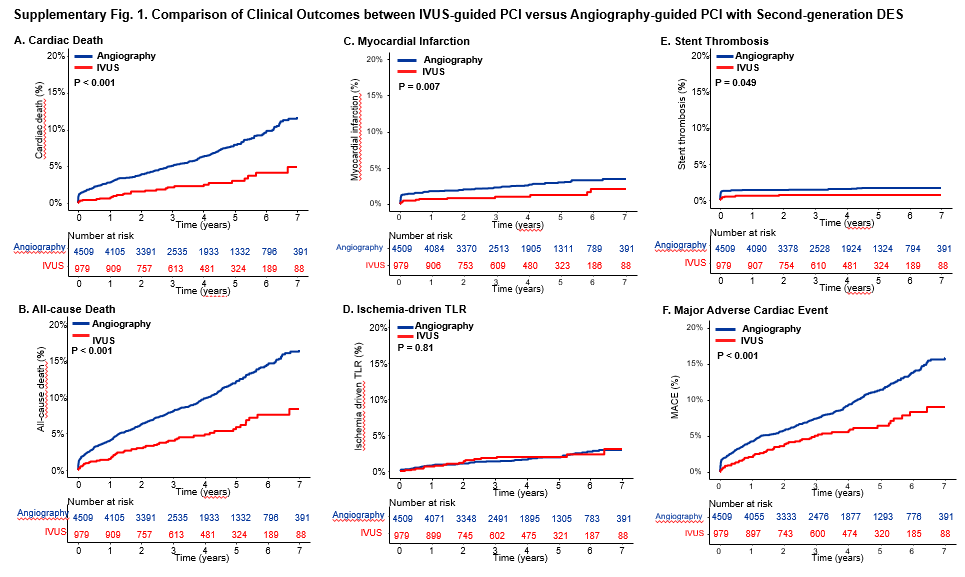


Kaplan-Meier curves are shown for comparison of risks of cardiac death (A), all-cause death (B), myocardial infarction (C), ischemia-driven TLR (D), stent thrombosis (E) and MACE (F) in the overall population.

Abbreviations: IVUS, intravascular ultrasound; DES, drug-eluting stent; MACE, major adverse cardiac event; TLR, target lesion revascularization.

**Supplementary Fig. 2. Landmark Analysis at 1-year after the Index Procedure**


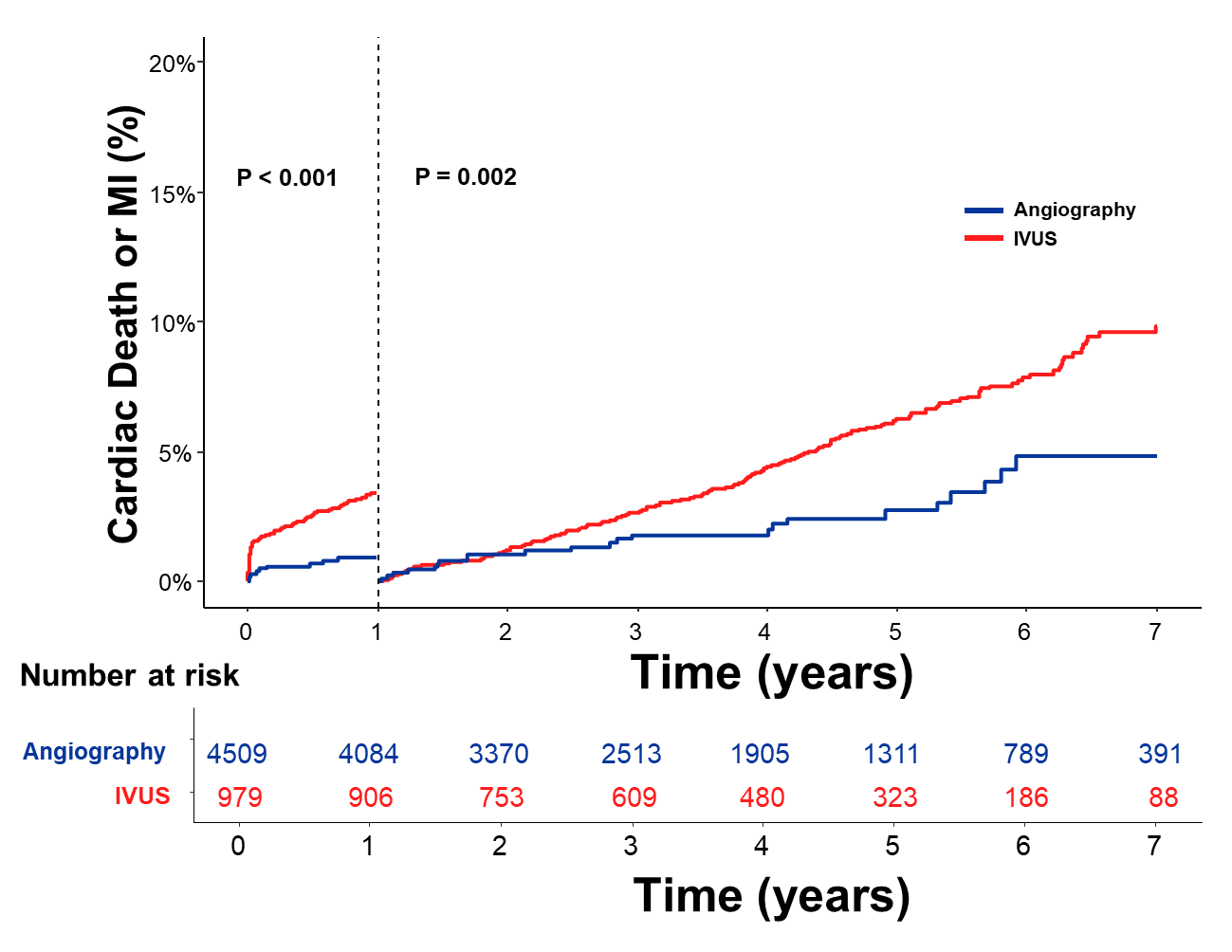


Kaplan-Meier curves are shown for comparison of risk of cardiac death or MI between IVUS-guided and angiography-guided PCI. The dashed line corresponds to the 1-year time-point for landmark analysis.

Abbreviations: IVUS, intravascular ultrasound; MI, myocardial infarction.
